# Addressing the Complexity of Tourette's Syndrome through the Use of Animal Models

**DOI:** 10.3389/fnins.2016.00133

**Published:** 2016-04-08

**Authors:** Ester Nespoli, Francesca Rizzo, Tobias M. Boeckers, Bastian Hengerer, Andrea G. Ludolph

**Affiliations:** ^1^Competence in Neuro Spine Department, Boehringer Ingelheim Pharma GmbH & Co. KGBiberach an der Riss, Germany; ^2^Department of Child and Adolescence Psychiatry/Psychotherapy, University of UlmUlm, Germany; ^3^Institute of Anatomy and Cell Biology, University of UlmUlm, Germany

**Keywords:** tics, repetitive behavior, genetics, environment, PPI, TS comorbidities

## Abstract

Tourette's syndrome (TS) is a neurodevelopmental disorder characterized by fluctuating motor and vocal tics, usually preceded by sensory premonitions, called premonitory urges. Besides tics, the vast majority—up to 90%—of TS patients suffer from psychiatric comorbidities, mainly attention deficit/hyperactivity disorder (ADHD) and obsessive-compulsive disorder (OCD). The etiology of TS remains elusive. Genetics is believed to play an important role, but it is clear that other factors contribute to TS, possibly altering brain functioning and architecture during a sensitive phase of neural development. Clinical brain imaging and genetic studies have contributed to elucidate TS pathophysiology and disease mechanisms; however, TS disease etiology still is poorly understood. Findings from genetic studies led to the development of genetic animal models, but they poorly reflect the pathophysiology of TS. Addressing the role of neurotransmission, brain regions, and brain circuits in TS disease pathomechanisms is another focus area for preclinical TS model development. We are now in an interesting moment in time when numerous innovative animal models are continuously brought to the attention of the public. Due to the diverse and largely unknown etiology of TS, there is no single preclinical model featuring all different aspects of TS symptomatology. TS has been dissected into its key symptomst hat have been investigated separately, in line with the Research Domain Criteria concept. The different rationales used to develop the respective animal models are critically reviewed, to discuss the potential of the contribution of animal models to elucidate TS disease mechanisms.

## Introduction

### TS definition, epidemiology, symptoms, and natural course

Tourette's Syndrome (TS) was named after Georges Gilles de la Tourette (1857–1904) who first described it as a “tic syndrome” in 1885 and whose observations are still considered mostly valid today. Tics are involuntary movements or vocalizations that can involve different parts of the body changing in frequency, intensity and duration. A diagnose of TS requires the presence of both multiple motor and one or more vocal tics with an onset before age 18 years and a persistence for at least 1 year (DSM-5).

But TS is not only about tics: up to 90% of all TS patients experience psychiatric comorbidities, mainly Attention Deficit/Hyperactivity Disorder (ADHD) and obsessive compulsive disorder (OCD), but also depression, anxiety disorders, conduct disorders, personality disorders, and self-injurious behaviors (Khalifa and Knorring, 2007; Cavanna et al., 2009; Pallanti et al., 2011).

TS has long been considered to be rare, as it was reported to affect only 1 in 2000 (Bruun, 1984). Nowadays the prevalence of TS in the general population has been re-evaluated, and is estimated to be 0.4–1% (Robertson et al., 2009), but could be even higher since, especially in childhood, tics are often so mild that are hardly perceived and easily overlooked. In many cases only an expert eye is able to identify tics in patients presented to the clinician as a consequence of behavioral problems or ADHD.

#### Role of CSTC circuitry in TS pathophysiology

The exact neurobiological background of TS remains still unclear, but a central role of the cortico-striato-thalamo-cortical (CSTC) circuit appears uncontroversial, as numerous anatomical and functional imaging studies were able to detect morphological and functional alterations in CSTC components of TS patients compared to controls (Singer et al., 1993; Peterson et al., 2003; Sowell et al., 2008).

The pre-motor and motor cortices, the striatum, composed of caudate and putamen, the globus pallidus internus (GPi) and externus (GPe), the subthalamic nucleus (STN), the thalamus, and the substantia nigra (SN) are connected in the CSTC circuit. Under physiological circumstances, an activation of this circuit physiologically results in voluntary movements, while involuntary movements are repressed.

Movements occur as the motor cortex is activated by the thalamus, which is controlled by the STN-GPe-GPi microcircuit. When the pre-motor cortex activates the putamen, the inhibitory striatal projection neurons release the thalamus from inhibition held by the STN-GPe-GPi, and eventually the motor cortex can be activated, leading to movement (Obeso and Lanciego, 2011).

Tics are supposed to be caused by a deregulated activity of the basal ganglia, which consequentially leads to disinhibition of the thalamus and a hyperexcitability of the motor cortex (Albin and Mink, 2006; Wang et al., 2011).

**Figure 1:**
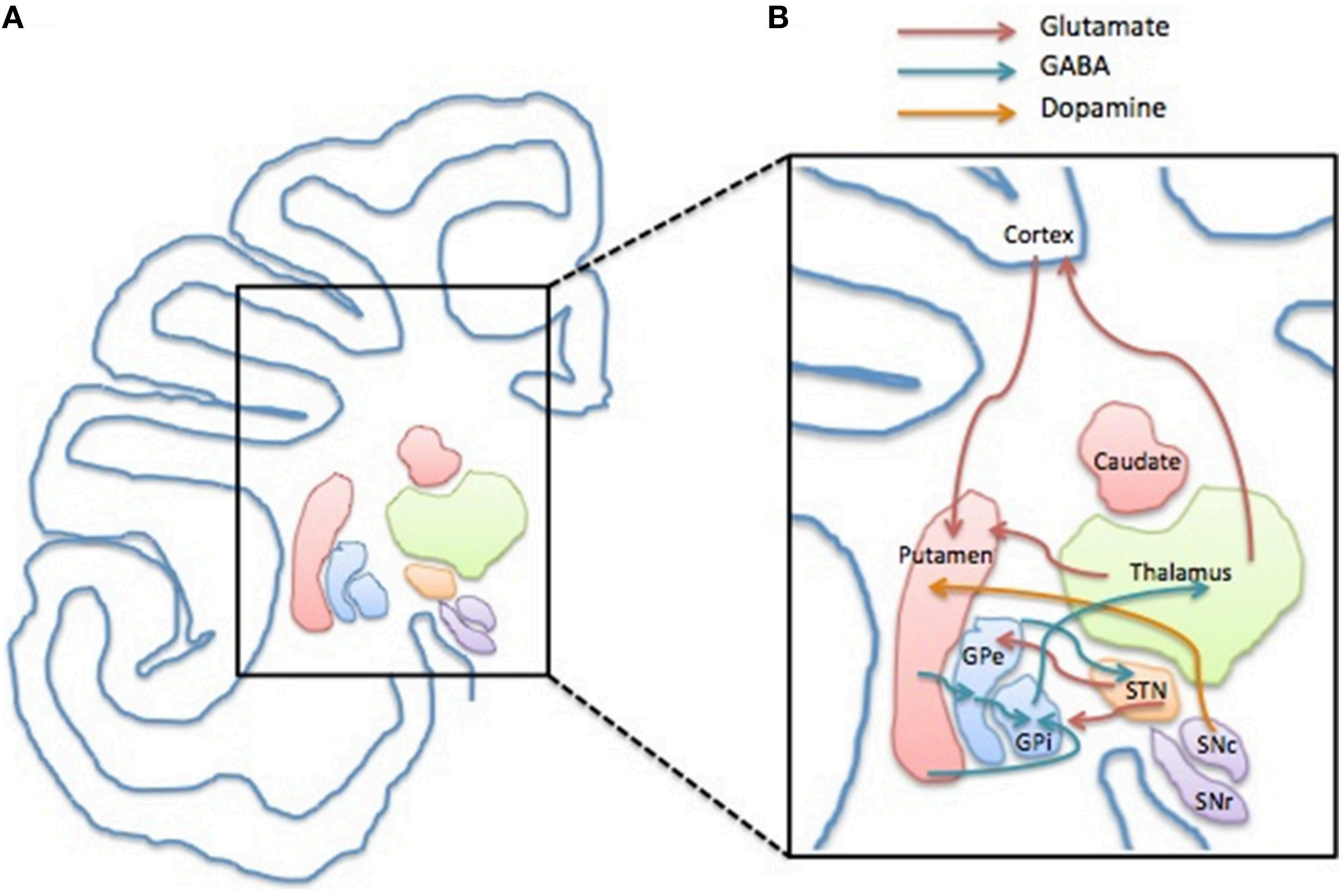
**Structure and compartments of CSTC circuit**. **(A)** Representation of a left side of human brain coronal section depicting the anatomical localization of the basal ganglia components and the cerebral cortex. **(B)** Schematic illustration of the CSTC internal network within the circuit depicting the glutamatergic (red arrow), GABAergic (blue arrow) and the dopaminergic connection (yellow arrow). Abbreviations: GPe, Globus Pallidus pars externa; GPi, Globus Pallidus pars interna; STN, Nucleus Subtalamicus; SNc, Substantia Nigra pars compacta; SNr, Substantia Nigra pars reticulata.

When the beneficial effect of dopaminergic modulators such as haloperidol and pimozide on tic management was observed, a dysfunction in the dopaminergic system was seen as the main responsible of TS neuropathology (for review see Buse et al., 2013). Nowadays the use of haloperidol and pimozide has been gradually left aside in favor of the better tolerable atypical antipsychotics and atypical neuroleptics, such as aripiprazole or risperidone, acting on dopamine and serotonin. In general, there is growing evidence indicating that TS is not a pure DA-related disorder, and the interplay of other neurotransmitters is strongly supported to contribute or cause the disease (for review see Udvardi et al., 2013; Figure 1).

#### Importance of a TS animal model

*In vivo* animal models are important tools to challenge and validate pathophysiological hypotheses and test new therapeutic options. An animal model is constructed to fulfill one or more of the following parameters: *face validity* (ability to show similar symptoms to the patients' ones), *construct validity* (model developed according to a rationale matching the pathological hypothesis), and *predictive validity* (model responds to a treatment similarly to patients). The ideal model is able to show all these three features, but in most cases the main focus remains on one of the three aspects. The use of animal models could help the major means of investigations of TS thanks to their ability to verify pathophysiological hypotheses and test pharmacological compounds.

## Methods

This article is a review about the “*now-in-use”* preclinical models of TS, extracted from the literature of the last decade. As a perfect model for TS has not yet been produced, we aim at showing the different successful methods used by researchers to independently model all major aspects involved in TS pathology, that we separately describe and analyze. Strengths and limitations of animal models are explained with a focus on recent research findings. The aim is to provide up-to-date information on TS animal models for students, researchers, and clinicians, and hints to be used by preclinical experimenter in developing new TS animal models.

Electronic literature search via MEDLINE/PubMed has been conducted for articles that had been published in English since year 2000. Combinations of keywords were used to identify relevant articles, including: “Tourette Syndrome,” “TS animal model,” “TS *in vivo*,” “motor tic,” “stereotype,” “premonitory urge,” “PPI,” “genetic TS,” “environment TS,” “immune TS,” “ADHD,” “TS neurobiology,” “OCD.” Systematic and narrative reviews, as well as original research articles were included. The last search was conducted on November 2015. The literature search was also supplemented with key publications and book chapters known to the authors.

## TS phenomenology

### Genetics

TS has a strong genetic basis. Family studies in children with TS reveal that 8–57% of their parents had a history of tics, and first-degree relatives had a significant increased risk of developing the disorder (Pauls et al., 1991). Twin studies also report a 53–56% concordance rate for TS in monozygotic twins, compared with only 8% in dizygotic twins (Price et al., 1985; Hyde et al., 1992).

The initial idea of TS being a monogenic Mendelian disorder has been quickly revised and TS is now considered a complex disorder with many open questions regarding its overall genomic architecture. The identification of TS-related genes through linkage and association studies is hindered by the unclear mode of inheritance, the genetic heterogeneity of the disease and its apparently incomplete penetrance (Pauls, 2003).

Specific genetic abnormalities have so far been identified in less than 1% of patients, including polymorphisms and copy number variation. Many of these findings also parallel those of other common neuropsychiatric and neurodevelopmental disorders, unveiling previously unknown disease mechanisms, but their specific role for TS has rarely been elucidated (Sundaram et al., 2010; Crane et al., 2011; Scharf et al., 2013; Bertelsen et al., 2015).

#### Modeling TS genetics

Animal genetic manipulation has widely been a key starting point to model numerous diseases.

Sequence variants in *Slirtk 1* were found in TS patients and associated to loss of function in supporting dendritic growth during development of numerous components of CSTC circuit (Abelson et al., 2005). *Slitrk1* KO mice exhibit elevated anxiety- and depression–like behaviors, symptoms which have also been associated withTS-spectrum disorder (Katayama et al., 2010).

The discovery of a mutation in the histidine decarboxylase (*Hdc*) gene in a unique family with marked history of tic disorders lead to the investigation of the disruption of histaminergic pathway in animal models. The core phenomenology of TS, tic-like behaviors, are not observed in *Hdc* KO mice at baseline, but stereotypies as repetitive sniffing and orofacial movements can be elicited by activating the dopamine system with D-amphetamine and are ameliorated after intracerebral administration of dopamine antagonist haloperidol. Fear conditioning significantly increased grooming in these animals (Castellan Baldan et al., 2014)^1^.

Furthermore, significant pre-pulse inhibition (PPI) deficits and striatal dopamine dysregulation have also been observed in *Hdc* KO mice, aligning human findings and supporting the interplay between histamine and dopamine, the major known player in TS (Rapanelli et al., 2014; Xu et al., 2015a).

Another recent genetic TS animal model has been developed based onthe observation that cholinergic interneurons are reduced by 50% in TS patient's striatum (Kataoka et al., 2010; Lennington et al., 2014): region-specific knockout of choline acetyltransferase in the dorsolateral striatum led to stress-induced increase in grooming. D-amphetamine administration did not increase the amount of grooming activity, but the animals performed more repetitive stereotyped actions (Xu et al., 2015b)^2^.

A main regulator of striatal activity is dopaminergic system whose alterations have been correlated with TS severity and the development of comorbidities. Genetic manipulation has been used as tool to address dopaminergic contribution to the pathology, even though genetic evidence for dopaminergic dysfunction has not been found in TS patients yet. Dopamine transporter (*DAT*) KO mice (Berridge et al., 2005) and dopamine receptor 3 (*DR3*) KO mice (Garner and Mason, 2002) are characterized by a hyperdopaminergic condition and show stereotypies, consolidating their role in repetitive behavior. Furthermore, *DAT* KO mice show a more complex and rigid sequence of actions during grooming, which is in between tics of TS and compulsions of OCD.

The lack of a clear, spontaneous “ticcing” phenotype in these genetic animal models raises the question of further neurotransmitters, synaptic, or developmental mechanisms that need to be evaluated (Table 1).

**Table 1:** **Genetic animal models of TS**.

**Transgenic Model**	**Gene target**	**Association to TS**	**Phenotype**	**References**
Slitrk1 KO mouse	SLIT and NTRK-like protein1	Slitrk1 mutated variants	Anxiety-like and depression-like behavioral abnormalities attenuated by clonidine (α2 adrenergic agonist) treatment	Katayama et al., 2010
HDC-KO mouse	Histidine Decarboxylase	HDC nonsense mutation	Increased grooming after D- amphetamine (5-HTR agonist) administration or stress. Stereotypies in HDC KO mice are mitigated by haloperidol (DA agonist) pretreatment	Castellan Baldan et al., 2014
ChAT-ablated mouse	Choline acetyltransferase	Reduced cholinergic interneurons in striatum of TS patients	No tic-like stereotypies and PPI deficit at baseline; increased and fragmented grooming after acoustic startle stimuli; increased stereotypies after amphetamine (5-HTR agonist) administration	Xu et al., 2015b
DAT-KO mouse	Dopamine transporter	–	Hyperdopaminergia in striatum and supestereotypies.	Berridge et al., 2005
			DA/5-HT imbalance in basal ganglia	Pogorelov et al., 2005
			PPI deficits and perseverative motor patterns	Ralph et al., 2001
DRD3-KO mouse	Dopamine receptor D3	–	Increase in spontaneous stereotypies	Garner and Mason, 2002
			Hyperlocomotor activity after amphetamine (5-HTR agonist) treatment	McNamara et al., 2006
DRD3-KO rat		–	Hyperactivity and rotational behaviors	

*List of TS animal models obtained through genetic manipulation. Note that not all human genetic mutations known to have a role in TS have been used to create a valid TS preclinical model. On the other hand, several transgenic animal models have shown a TS-related phenotype but no correlation with a known TS mutation has been found so far. Abbreviations: 5HT2c-KO, Serotonin receptor knock out; CNTNAP2, Contactin-associated protein-like2; COL27A1, Type XXVII collagen alpha chain gene; CRL, controls; DAT-KO, Dopamine transporter knock out; DAT-KD, Dopamine transporter knock down; DRD1-KD, Dopamine receptor D1 knock down; DRD3-KO, Dopamine receptor D3 knock out; GABRB3, GABA A-receptor beta-3; HDC, Histidine decarboxylase; IMMP2L, Inner mitochondrial membrane peptidase; NLGN4X, Neuroligin-4 protein; POLR3B, polymerase (RNA) III (DNA directed) polypeptide B; PPI, Pre-pulse Inhibition; SLITRK1, SLIT and NTRK-like protein1; 5-HTR, serotonin receptor*.

### Tics

A tic is a sudden, rapid, recurrent, non-rhythmic, jerk-like movement, or vocalization that can vary in frequency, intensity, duration and anatomical localization. Tics are classified as simple or complex according to the number of groups of muscles involved, and as motor or vocal tics.

Simple tics usually last few milliseconds engage one or a group of muscles like those involved in eye blinking (simple motor tic) or throat clearing (simple vocal tics). Complex tics last few seconds and can be defined as a combination of simple tics. They can appear purposeful like performing obscene gesture (copropraxia) or uttering racial slurs (coprolalia) or may consist in the imitation of someone elses′ actions (echopraxia) or words (echolalia).

Three different tic disorders are included in the DSM-5*:* provisional tic disorder, persistent motor, or vocal tic disorder and TS. The difference between these disorders relies on the type of tics observed (motor, vocal, or both), and how long the symptoms have lasted. The presence of both motor and vocal tics for a period longer then 1 year since first onset (before 18 years of age) and their “waxing and waning” course differentiate TS. Indeed, they may show a pattern in which old and new tics overcome and fluctuate in frequency and intensity over time.

Other hyperkinetic movements can occur in TS patients and can be easily misdiagnosed and lead to a wrong treatment approach (Kompoliti and Goetz, 1998). This is the case of stereotypies that are fixed, prolonged and rhythmic repetitive behaviors and present an average age of onset of 3 years (DSM-5). Unlike stereotypies, tics are tipically preceded by an uncomfortable phenomenon called “premonitory urge” (PU) and can be voluntarily suppressed by most patients for a short period of time.

In general, tics are intensified by stress, anxiety, excitement, anger, fatigue, or infections (Lombroso et al., 1991; Nelson, 1993; Lin et al., 2007) while their reduction is reported in patients performing focused and effortful activities (Conelea and Woods, 2008).

#### Modeling Tics

The clear terminology available for clinicians to identify motor disorders is not easily applicable by *in vivo* experimenters, as any parallelism between human and animal condition must be taken carefully.

Literature testifies the lack homogeneity employed to name motor phenotypes in animal models of TS, ranging from “tic,” to “tic-like movement,” or “repetitive movements” and “stereotypies.”

Several animal models of tics have been obtained through systemic or focal administration of active substances, which give a transient but easy to replicate phenotype. Importantly, different compounds with diverse effects were proven to be effective in the induction of tic-like behavior.

The intracerebral infusion of GABAergic antagonists is becoming a more and more appealing strategy of tic-like movement induction and has led to the formulation of the hypothesis that disequilibrium between cortical glutamatergic output and striatal GABAergic metabolism plays an important role in tic induction. The GPe was one of the first basal ganglia components to be investigated with this approach (Grabli et al., 2004), but is now the functional disruption of the striatum to be the major target of investigation.

Striatal injections of the GABAergic antagonist bicuculline in primates cause simple tic-like movements, hyperactivity and stereotyped behaviors (McCairn et al., 2009). These three phenotypes are independent processes and appear to be associated with different brain regions: the sensorimotor network, the prefrontal cortex and associative territories and the orbitofrontal cortex and limbic part of the basal ganglia respectively (Worbe et al., 2013). Electrophysiological data also suggest a role for the cerebellum in tic expression in this model (McCairn et al., 2013). The application of the same approach in adult rats results in an acute tic session that varies in intensity and body parts involved and is characterized by additional hyperactivity (Bronfeld et al., 2013)^3^. In mice, tics were also evoked by striatal picrotoxin injections, while cortical injections induce seizures (Pogorelov et al., 2015)^4^.

Systemic administration of hallucinogens acting on serotonin receptors (Tizabi et al., 2001; Fantegrossi et al., 2005, 2006; Halberstadt and Geyer, 2014; Ceci et al., 2015) induce head-twitches responses, while the use of monoamines modulators, induces stereotypies (Lv et al., 2009; Taylor et al., 2010). Stereotypic behaviors were also observed after administration of 3,3′-iminodipropionitrile (IDPN) (Wang et al., 2013) and *Catha edulis extract* (Oyungu et al., 2007).

The D1CT-7 transgenic mouse, originally proposed for OCD, shows head twitching and abnormal movements of limbs and trunk with juvenile onset and sexual dimorphism (Nordstrom and Burton, 2002). These animals display PPI deficits and tic-like manifestations that are increased in presence of spatial confinement-induced. This model appears to show higher hyperactive stress reduced by antipsychotics and clonidine (Nordstrom et al., 2015), making it the first model to show *face validity* for tics and feature also common TS-related phenotypes (Table 2).

**Table 2:** **Animal models of tics**.

**Approach**	**Method**	**Compound**		**Phenotype**	**References**
Pharmacological	Systemic injection	Hallucinogens (5HTR agonists)	DOI in mice	Head twitch response. Reduced by donepezil (acetylcholinesterase inhibitor), nicotine (nAChR agonist) and haloperidol (DA antagonist) chronic or acute treatment	Hayslett and Tizabi, 2003; Tizabi et al., 2001
			DOI in ABH, C57BL/6N, SJL/J, and CD-1 mice	Head twitch response and skin jerk responses. URB597 (FAAH inhibitor) reduced head twitch in all strains	Ceci et al., 2015
			2C-I in mice	Head twitch response. Blocked by M100907 (5-HTR antagonist) administration	Halberstadt and Geyer, 2014
			2C-T-7 in mice	Head twitch response. Antagonized by M100907 (5-HTR antagonist)	Fantegrossi et al., 2005
			5-MeO-DIPT in mice	Head twitch response. Antagonized by M100907 (5-HTR antagonist) pretreatment	Fantegrossi et al., 2006
			Metamphetamine-induced hyperactive mice	Motor tics and hyperactivity. Reduced by hispidulin (plant extract with antiepileptic activity) pretreatment	Huang et al., 2015
		Dopamine modulators	Apomorphine in rats	Stereotyped actions. Inhibited by ningdong (biological extract) and haloperidol (DA antagonist) treatment	Lv et al., 2009
			SKF38393 in rats	Super-stereotyped syntactic grooming chain. Ameliorated by haloperidol (DA antagonist)	Taylor et al., 2010
		Others	IDPN (neurotoxin) in mice	Stereotypies ecresaed by tiapride (DA antagonist) and by Jian-Pi-Zhi-Dong Decoction (plants extracts)	Wang et al., 2013
			Khat cathinone (*Catha edulis extract*) in rats	Seizures, stereotyped behaviors	Oyungu et al., 2007
	Focal and systemic injection	Hallucinogens in frontal cortex of wild type and B-arr2 KO mice		Head twitch response	Schmid and Bohn, 2010
	Focal injection	GABA antagonists	Picrotoxin injections in DLS and SMC of mice	Injections in DLS induced tic-like movement attenuated or abrogated by PMPA (NMDAR antagonist) and muscimol (GABA agonist) pretreatment; injecitons in SMC produced tic-like movements and hyperactivity abrogated by muscimol pretreatment	Pogorelov et al., 2015
			BIM injections in rat GPe	Stereotypies, attention deficits and hyperactivity	Grabli et al., 2004
			BIM injections in rat striatum	Tic movements somatotopically organized and hyperbehavioral abnormalities	Bronfeld et al., 2013
			BIM injections in primate striatum	Periodic orofacial tics and forelimb tics, hyperactivity and stereotypic behaviors. Tics did not interfere with overall normal behavior	McCairn et al., 2009; Worbe et al., 2013
Genetic	D1CT-7 transgenic mice	–		Seizures, tics and compulsive behaviors increased by pentylenetetrazol (convulsivant)	Campbell et al., 2000
		–		Repetitive climbing and leaping. Dizocilpine (non-competitive NMDAR antagonist) aggravated the phenotype and induced seizures; NBQX (AMPAR blocker) reduced stereotypies and seizures.	McGrath et al., 2000
		–		Tic-like movements; sensorimotor gating deficit in response to spatial confinement	Godar et al., 2015

*List of animal models that show a motor phenotype that can be related to tic spectrum as predominant and relative drugs treatment approaches. Phenotypes are indicated as reported in literature. Abbreviations: BIM, bicuculline methiotide; DLS, dorsolateral striatum; DOI, 2,5-Dimethoxy-4-iodoamphetamine; FAAH, fatty acid amide hydrolase; IDPN, 3,3′-iminodipropionitrile; M100907, (R)-(+)-α-(2,3-dimethoxyphenyl)-1-[2-(4-fluorophenyl)ethyl]-4-piperidinemethanol; NBQX, (2,3-dihydroxy-6-nitro-7-sulfamoyl-benzo[f]quinoxaline-2,3-dione); nAChR, nicotinic acetylcholine receptor; PMPA, (RS)-4-(phosphonomethyl)piperazine-2-carboxylic acid; SMC, sensorimotor cortex; URB597, fatty acid amide hydrolase; 2C-I, 2; C-I (2,5-dimethoxy]]-4-iodophenethylamine; 2C-T-7, 4-propylthio-2,5-dimethoxyphenethylamine; 5-MeO-DIPT, 5-Methoxy-diisopropyltryptamine; 5-HTR, serotonin receptor; 5-HTTP, 5-hydroxy-L-tryptophan*.

### Premonitory urge

Since pediatric age, TS patients become aware of an uncomfortable sensation that precedes tics known as premonitory urge (PU) that, for about 57% of cases, is more bothersome then tics themselves (Cohen and Leckman, 1992; Reese et al., 2014).

From a therapeutic point of view, the understanding of PU might help tic management since it could enhance the patient's own ability to suppress it (Leckman et al., 1993; Frank and Cavanna, 2013).

In adult TS patients the neurophysiological system of urge and tic generation appears to be distinct from the one implied in tic control (Ganos et al., 2012): the urge would include both voluntary motor circuits and somatic sensation circuits (anterior cingulate cortex and supplementary motor area), while tic generation is known to take place in prefrontal structures involved in the primary inhibition of the motor control, as confirmed by neuroimaging studies (Peterson et al., 1998).

The genesis of PU is still unknown but some evidence led to the hypothesis that this feeling might reflect abnormalities of sensorimotor gating, i.e., the neurological process able to filter out redundant or unnecessary environmental stimuli that constantly reach our brain (Braff et al., 2001; Biermann-Ruben et al., 2012).

#### Modeling TS sensorimotor gating deficit

Tics are, to a certain extent, an easy-to-detect phenomenon; PU is more complicated to be translated into a preclinical model but can be investigated through the study of sensorimotor gating deficit.

To assess sensorimotor gating functions, the pre-pulse inhibition (PPI) of the startle response is used in both humans and laboratory animals. PPI is a behavioral phenomenon in which a weak pre-stimulus (i.e., prepulse) diminishes the reaction to a subsequent stronger stimulus (i.e., pulse) that could otherwise trigger a strong startle response. In presence of acoustic, visual or tactile stimuli, TS patients show PPI deficits manifesting the inability to filter unnecessary information (Castellanos et al., 1996; Zebardast et al., 2013).

Due to its conformity to the validity criteria, this animal model of sensorimotor gating deficits has now reasonably been extended from the single research of schizophrenia (Wan and Swerdlow, 1996) to the study of TS and its comorbidities (Swerdlow and Sutherland, 2005). In rodents, PPI appears to be regulated by the nucleus accumbens and its dopaminergic activation. Similar to tics, PPI abnormalities develop in rats treated with dopaminergic agonists (Alsene et al., 2010; Mosher et al., 2015), hallucinogens (Swerdlow et al., 2003; Chen et al., 2012) and glutamate antagonists (Swerdlow et al., 2007; Pietraszek et al., 2009). PPI deficit could also be detected in spontaneous hypertensive rats (SHR), the model of choice for ADHD (Van Den Buuse, 2004; Table 3).

**Table 3:** **Animal models of PPI deficit: List of animal models that show a PPI deficit**.

**Approach**	**Method**	**Compound**	**Phenotype**	**References**
Pharmacological	Systemic administration	Metamphetamine (5-HTR agonist), ketamine, and dizocilpine (non-competitive NMDAR antagonists) in mice	PPI deficits alleviated by *Clerodendrum inerme* ethanol extract treatment	Chen et al., 2012
		Apomorphine (DA agonits), amphetamine (5-HTR agonist), and DOI (5-HTR agonist) in parental Sprague Dawley and Long Evans rats, and offspring	Strain related heritable PPI changes	Swerdlow et al., 2003
		Dizocilpine (non-competitive NMDAR antagonists) in rat	Locomotor hyperactivity, PPI disruption, working memory deficit not alleviated by 1MeTIQ (NMDAR antagonistic)	Pietraszek et al., 2009
		Amphetamine (5-HTR agonist) in rat	PPI deficit and hyperactivity. Blocked by prazosin (α1 adrenergic receptor blocker) and partially by terazosin (α1 adrenergic receptor antagonist) focal administration in nucleus accumbens	Alsene et al., 2010
		Dizocilpine (non-competitive NMDAR antagonists) or apomorphine (DA agonits) in rat	PPI deficit. Abolished by GTS-21 (AChR partial agonist) clozapine (5-HTR partial agonist) and haloperidol (DA antagonist)	Callahan et al., 2014
		SKF82958 (DA full agonist) in Sprague-Dawley, Wistar, and Long Evans rats	Strain-specific PPI deficits.	Mosher et al., 2015
	Focal administration	p-Hydroxyamphetamine (TAAR1 agonist) in mice	PPI deficit attenuated by pretreatment with 5,7-DHT (serotonin-containing neurons neurotoxin), PCPA (serotonin synthesis inhibitor), ketanserin (5-HTR antagonist), and MDL100,907 (5-HTR antagonist)	Onogi et al., 2011
		Granulocyte-Macrophage Colony-Stimulating Factor in rat	Hyperlocomotion; social interaction and PPI deficits. Alleviated by minocycline (antibiotic)	Zhu et al., 2014
	Systemic and focal administration	Apomorphine (DA agonits) in Sprague Dawley and Long Evand rats	PPI distrupted in Sprague-Dawley	Swerdlow et al., 2007
		Apomorphine (DA agonits) and amphetamine (5-HTR agonist) in rats	PPI deficit. Prevented by finasteride (5α-reductase inhibitor)	Devoto et al., 2012
Genetic	–	BTBR mice	Spontaneous stereotypic behavior	Pearson et al., 2011
	–	Wistar and SHR rat	SHR PPI lower then Wistar rats. Reversed by WIN55212,2 (CBR agonist) and cannabidiol (CBR indirect antagonist)	Levin et al., 2014
	–	HET mice	Behavioral and PPI deficits	Chohan et al., 2014
Environmental	Prolonged maternal deprivation in rats	–	PPI reduction and impaired spatial learning in adulthood	Garner et al., 2007
	Social isolation in rats	–	Increased self-grooming and locomotor activity, PPI deficit	Strauss et al., 2014
	Pre- and post- weaning maternal separation and social isolation in rats	–	PPI changes in the adults following maternal separation and not social isolation	Weiss et al., 2011

*Abbreviations: CBR, Cannabinoid receptor; DA, Dopamine; DOI, 2,5-Dimethoxy-4-iodoamphetamine; GTS-21, α7-nAChR agonists (also known as DMXB-A); HET, head tilt gene; KO, knock out; PPI, Pre Pulse Inhibition; PCPA, p-chlorophenylalanine; SHR, Spontanueous Hypertensive Rat; TAAR1, Trace amine-associated receptor 1; 1MeTIQ, 1-Methyl-1,2,3,4-tetrahydroisoquinoline; 5,7-DHT, 5,7-dihydroxytryptamine*.

### Environmental risk factors

Similar to other developmental neuropsychiatric disorders, TS perfectly fits in a so-called “multistrike model” of etiology. In this model the first hit is represented by the genetic vulnerability to the disease that is likely to be translated in structural and functional neurological changes. If these changes disturb regions with physiological self-regulatory functions -second hit- tic expression is evoked. In addition, various environmental factors (neuroendocrine, infectious, autoimmune, toxic, and psychosocial influences), representing a third strike, further increase the risk of tic expression (Spessot et al., 2004).

Numerous studies have investigated environmental factors that might contribute to the onset and severity of TS and associated comorbidities. Chao et al. (2014) systematically reviewed studies investigating the contribution of pre- and perinatal adverse events on onset and severity of TS and its comorbidities, if present.

Maternal smoking appears to be consistently implicated to TS pathology (Mathews et al., 2006; Motlagh et al., 2010).

Infections, and particularly Pediatric Autoimmune Neuropsychiatric Disorders Associated with Streptococcal Infections (PANDAS) were associated to worsening or causing TS (Kurlan, 2004; Kirkman et al., 2008; Singer et al., 2012; Swedo et al., 2012), however, a causal relationship between streptococcal infections and TS is still under investigation (Hoekstra and Minderaa, 2005; Krause et al., 2010).

Finally, only a few clinical studies were conducted investigating the extent to which stressors affect TS patients' life (Silva et al., 1995) but TS patients report a strong link between stress and tics exacerbation. The hypothalamic-pituitary-adrenal axis is supported to have an enhanced responsivity in children with TS (Corbett et al., 2008) and tic severity seems to correlate to cortisol levels (Conelea and Woods, 2008).

#### Modeling TS environmental risk factors

Several immune-mediated models have been developed according to different strategies.

Passive exposure to immunomediators (Ponzio et al., 2007; Smith et al., 2007; Depino et al., 2011; Patel et al., 2012; Zalcman et al., 2012) or to immunogenic microbial components (Hoffman et al., 2004; De Miranda et al., 2010; Yaddanapudi et al., 2010; Brimberg et al., 2012; Kirsten et al., 2012; Malkova et al., 2012) led to increased stereotypies and locomotion. However, additional deficits in motor coordination, learning/memory and social interaction, and the presence of immune deposits in the brain severely hamper their *face validity* for TS (Yaddanapudi et al., 2010).

Transplantation into naïve animals of antibodies derived from animals actively immunized with patients' sera (Taylor et al., 2002; Singer et al., 2005; Martin et al., 2008; Zhang et al., 2012) led to a similar phenotype and episodic vocalizations were reported (Hallett et al., 2000).

The importance of stress as a factor able to exacerbate tics has for long been referred by patients. Stress paradigms have proven capable of worsening the phenotype in animal models and have been recently introduced as a way to improve their validity (Xu et al., 2015a,b).

Stress paradigms can also be used to evaluate the ability of different stressors to predispose to abnormal behavioral development (Hall, 1998; Pryce and Feldon, 2003). For istance, maternal deprivation affects the social, emotional and attention domain of primates leading often to stereotypies or other dysfunctional motor activities (Márquez-Arias et al., 2010; Rommeck et al., 2011; Table 4).

**Table 4:** **Animal model of environmental factors influencing TS**.

**Approach**	**Method**	**Compound**	**Phenotype**	**References**
Immuno-mediation	Overexpression of brain immunemediators levels	Peripheral injection of IL-2 in rats during mid gestation	Stereotypic behaviors and decreased conditioned eye response	Ponzio et al., 2007
		Peripheral injection of IL-6 in mice during mid-gestation	PPI deficit	Smith et al., 2007
		Focal injection of TGFbeta-1 in mice hippocampus	Early: stereotypy behaviors, depression. Adult: decreased stereotypies and depression	Depino et al., 2011
		Peripheral injection of sIL-2R alfa/beta	Increased rearing, turning, grooming, head obbing, and jumping	Zalcman et al., 2012
		Focal injection of sIL-6R alfa	Hyper locomotor activity and stereotypic behaviors	Patel et al., 2012
	Auto-antibodies injections	Focal injection of IgG positive for antineuronal abs in rat striatum	Increased motor stereotypies and episodic vocalizations	Hallett et al., 2000
		Focal injection of anti-strep IgM mAb in mice.	Increased stereotypies, head bobbing, and grooming.	Zhang et al., 2012
		Focal injection of TS sera in rat striatum	Increased oral stereotypies and genital grooming	Taylor et al., 2002; Singer et al., 2005
		Peripheral injection of IgG from mothers of ASD children in the first trimester of pregnancy in primates	Increased stereotypies and hyperactivity	Martin et al., 2008
	Exposure to microbial immunogen or mimics.	Focal injection of GAS (M6-type) homogenate in mice	Stereotypic behavior, anxiety, and depression	Hoffman et al., 2004; Yaddanapudi et al., 2010
		Peripheral injection of GAS (M18 type) cell wall components in rats	Motor abnormalities and obsessive-compulsive behaviors. Alleviated by haloperidol (D_2_R antagonist) and paroxetine (SSRI), respectively	Brimberg et al., 2012
		Peripheral Poly I:C injection in mice during mid gestation	Increased grooming	Malkova et al., 2012
		Peripheral Poly I:C injection in mice during late gestation	Poor early motor coordination, PPI deficit, increased locomotor activity. Behavioral deficits reversed by carprofen (COX-2 inhibitor)	De Miranda et al., 2010
		Peripheral LPS injection in rats during mid gestation	Increased repetitive behaviors in male offspring	Kirsten et al., 2012
Stress	Differential raising conditions in primates	–	Stereotypies and SIB in nursery-raised group more than mother-raised and in the indoor raised group more then outdoor raised groups	Rommeck et al., 2011
	Environmen-tal enrichment Captive primates	–	Repetitive movements without paying attention to the surroundings, such as pulling one's hair, cheek pinching and swinging the body Stereotypies. Environmental enrichment reduces stereotypies, aggression and coprophilia and enhances exploration	Márquez-Arias et al., 2010

*List of animal models in which the TS-related phenotype is reached using environmental factor modification. Abbreviations: Ab, antibody; GAS, group A streptococcus; COX, cyclooxygenase; Ig, immunoglobulin; IL, interleukine; LPS, Lipopolysaccharide; mAb, monoclonal antibody; PPI, Pre-Pulse Inhibition; SIB, self-injury behavior; SSRI, selective serotonin re-uptake inhibitors; TGF, tumor growth factor; TS, Tourette's syndrome*.

### Related psychiatric conditions

#### ADHD

Attention-deficit/hyperactivity disorder (ADHD) is the most common comorbidity in TS.

ADHD is a neurodevelopmental disorder with an onset before age 12 (DSM-5). It affects about 5% of children, with 2–4:1 boys/girls prevalence (Polanczyk and Rohde, 2007). The three core symptoms of ADHD are inattention, motor hyperactivity, and increased impulsivity. Inattention refers to disorganization and difficulty in sustaining focus; hyperactivity manifests as excessive motor activity or talking activeness in inappropriate situations; impulsivity refers to the tendency to perform, without adequate forethought.

The cause of ADHD still remains elusive but it most likely results from a combination of cofactors that can be genetic, developmental, and/or environmental. The observation that the most effective drugs for ADHD treatment are psychostimulants (Sagvolden et al., 2005), implicates a role for catecholamines in the development of the disease. Indeed, the dopaminergic D1, D4, and D5 receptor genes, the α2-adrenoceptor gene, and both dopamine and norepinephrine transporters (DAT1, NET1) genes show polymorphisms in ADHD patients (Cook et al., 1995; Manor et al., 2004; Bobb et al., 2005; Park et al., 2005; Kickler et al., 2009). Serotonin has also been indicated to play a role in ADHD, as suggested by polymorphisms in genes that encode the serotonin transporter and the serotonin 1B receptor (Kent et al., 2002).

Since ADHD affects 60–80% of children with TS (Khalifa and Knorring, 2007), a common pathophysiological link between these two disorders seems evident. A debate is going on whether the two pathologies are independent (additive model), combined (interactive model), or a phenotype subgroup of one of the two major clinical forms (phenotype model) (Cavanna et al., 2009; Greimel et al., 2011; Schlander et al., 2011), however, there is increasing evidence for an additive model (Lebowitz et al., 2012; Roessner et al., 2007).

##### Modeling ADHD

Inattention, motor hyperactivity, and increased impulsivity are the three core features of ADHD. They have been differently modeled using (i) genetic manipulation, for instance in DAT-KO mice, coloboma mutant mice, nicotinic receptor mutant mice, human thyroid receptor expressing mice, GAT1-KO mice, ACC mice, and mutant tachinin-1 mice (Gainetdinov and Caron, 2000; Granon and Changeux, 2006; Siesser et al., 2006; Bruno et al., 2007; Yan et al., 2009; Zimmermann et al., 2014), (ii) selective breeding, as in SHR rats and Naples high excitability rats (Sadile et al., 1993; Sagvolden, 2000) (iii) insulting events during early developmental stages through 6-hydroxydopamine lesion and prenatal nicotine exposure (Stead et al., 2006; Schneider et al., 2011; Zhu et al., 2012; Freund et al., 2014) (iv) social isolation (Ouchi et al., 2013).

To validate these models, sustained attention deficits should be shown when stimuli are widely spaced in time, hyperactivity should be absent in novel situations and develop gradually over time and impulsivity should be sensitive to reinforcers (for review see Sagvolden et al., 2005).

SHR rats have been the most extensively used model of ADHD and feature all core aspects of this disorders. However, in SHR rats and in all previously listed ADHD models tic-like behaviors have not been documented.

Animal models of TS showing comorbid full ADHD spectrum have not been reported so far, but some validity for the single features were documented: hyperactivity was associated to specific bicuculline injections sites in the dorsal striatum and dorsal GPe of primates (Grabli et al., 2004; Worbe et al., 2009) and attention deficit occurred after injections in associative regions of the GPe (Grabli et al., 2004).

#### OCD

OCD is a neuropsychiatric disease that is frequently found as comorbidity in adult TS patients. It is a chronic disorder, which affects approximately 1–3% of the population (Pallanti et al., 2011).

According to DSM-5, obsessions, compulsions, or both, have to be present for an OCD diagnosis. Obsessions are defined as recurrent and persistent thoughts (e.g., fear of contamination), urges (e.g., need to wash hands), or images (e.g., of a violent or horrific scene) that are experienced as intrusive and unwanted, and cause marked anxiety and distress. The individual will try to suppress or to neutralize obsessions with some other thoughts or actions, for instance by performing a compulsion. Compulsions are defined as repetitive mental acts (e.g., counting) or behaviors (e.g., washing hands) performed in response to an obsession or according to rules that must be applied rigidly to a clearly excessive point when they become disruptive for daily living. OCD patients are able to recognize their obsessions and compulsions, but are unable to avoid them (Koran et al., 1996; Okasha et al., 2000).

The etiology of OCD is not completely understood.

Serotonin was the first neurotransmitter to be associated with OCD pathophysiology when selective serotonin re-uptake inhibitors (SSRIs) were shown to be efficacious in treating OCD (Barr et al., 1992). However, many patients do not respond to SSRIs treatment suggesting the additional involvement of other NTs such as dopamine (Carey et al., 2005; Taj et al., 2013), GABA (Simpson et al., 2012; Russo and Pietsch, 2013; Russo et al., 2014) and particularly glutamate (Arnold et al., 2006; Alonso et al., 2012; Porton et al., 2013). Growing evidence indicates the latter as a putative central player in OCD pathophysiology, strengthening the glutamate hypothesis of OCD and opening a new window for the development of novel treatment strategies (Coric et al., 2005; Grant et al., 2007; Bakhla et al., 2013).

Dopamine, GABA, and glutamate are commonly associated to CSTC circuit malfunction, implicating a role for this circuit in OCD pathophysiology (Stahl, 1988; Insel and Winslow, 1992; Graybiel and Rauch, 2000; Welch et al., 2007). Such alterations are also thought to be causative of tics, which 30% of OCD patients develop (Bloch et al., 2006; Pallanti et al., 2011). Tics and compulsions are now considered to be two different sides of the same coin that may be grouped under the general term of “tic-like” activities (Lombroso and Scahill, 2008; Worbe et al., 2010; Cath et al., 2011; Martino et al., 2013).

##### Modeling OCD

In animal models reported to have validity for OCD the presence of obsessions has been reasonably left aside and the focus was on the presence of behavioral compulsivity, intended as the performance of repetitive, and perseverating actions and stereotypies (for review see Alonso et al., 2015).

It is interesting to underline the existence of an analog to OCD in dogs: the Canine Compulsive Disorder (CCD), which leads to excessive tail chasing, light/shadow chasing, and flank sucking. These behaviors are attenuated with the same treatments used for OCD, indicating that its study may help elucidate the etiology of compulsive disorders (Ogata et al., 2013).

Numerous validated approaches have been developed aiming to evaluate and quantify compulsive-like behaviors. Examples are the schedule-induced polydipsia (Woods et al., 1993), the marble burying test (Ichimaru et al., 1995), the signal attenuation test (Joel et al., 2005) the nest building test (Hoffman and Rueda Morales, 2012) and the nestlet shredding test (Angoa-Pérez et al., 2012). These models provide the greatest ease of use and do not require any pharmacological or genetic intervention but on the other hand they do not offer any pathophysiological information.

Based on the clinical evidence for an involvement of serotonin in OCD, OCD-like behaviors are induced in animals by treatments with serotonergic agonists 8-hydroxy-2-(di-n-propylamino) tetralin (8-OH-DPAT) (Carli et al., 2006; Arora et al., 2013) and m-chlorophenylpiperazine (mCPP) (Kreiss et al., 2013), as well as with the serotonin releasing agent compound 48–80 (Wald et al., 2009). Mice lacking TPH2, the rate-limiting enzyme of serotonin synthesis in the brain, display highly repetitive and compulsive behaviors (Kane et al., 2012).

The glutamatergic hypothesis of OCD finds also a strong support in animal models. In mice lacking the AMPA receptor trafficking protein SAPAP3, glutamate signaling dysfunction is accompanied by compulsive grooming behavior (Welch et al., 2007; Wu et al., 2012).

Astrocyte-specific glutamate transporter (GLT1) inducible knockout mice exhibit OCD/TS-like behavioral spectrum, with marked increased self-injurious grooming behavior (Aida et al., 2015). Interestigly, this is the first hint of a role for non-neuronal cells in this brain disorder. Lastly, transmembrane protein *Slitrk5* KO mice show OCD-like behavioral abnormalities that seem to be associated to a deficient corticostriatal neurotransmission (Abelson et al., 2005; Shmelkov et al., 2010). *Slitrk5* belongs to the same family of *Slitrk1*, a protein associated to TS.

Dopamine, that has been largely associated to TS and ADHD, is supported by animal models findings to play a role in compulsive behaviors. The treatment with the DR2 agonist quinpirole in mice marks the expression of the behavioral repertoire and long-term exposure to this drug leads to hyperactivity in A/J mice (De Haas et al., 2012). In rats, chronic administration of the same compound causes compulsive checking (Szechtman et al., 1998; Alkhatib et al., 2013).

In the D1CT-7 transgenic mouse, the modulation of the glutamatergic cortical output on the striatal circuits obtained through the chronic potentiation of cortical and limbic D1-expressing neurons leads to the development not only of compulsive behaviors, but also of tics. This makes it the only model of comorbid tics and OCD proposed so far (Smicun et al., 1999; Nordstrom and Burton, 2002).

## Conclusions

Animal models are gaining an important role in understanding TS pathophysiology and in investigating new treatment options. In the recent years numerous models have been developed, many of which summarize more than a single aspect of the syndrome.

Through animal models the idea of a major role for the striatum in tics generation, already suggested by imaging and *post mortem* studies, was importantly strengthened. In fact, independent approaches used to model TS succeeded in showing increased grooming and tic-like phenotype following striatal structural and functional alterations. This indicates the striatum as a research target worth investing more efforts.

Reproducing tics, the core feature of TS, is the actual greatest challenge for animal models. A TS diagnosis requires the coexistence of multiple motor and at least a vocal tic, but so far researchers focused on motor tics while the presence and cause of vocal tics has been poorly investigated and require further attention.

The difference between tics and other movement disorders can be detected in humans but it is subtler in animals that physiologically account a wide range of species-specific repetitive movements in their repertoire. In patients, tics have the peculiar features of being preceded by a PU, have a waxing and waning pattern and can be voluntarily repressed. These distinctive features of tics are difficult to observe in animals and result severely biased by the approach used. Stereotypies, which are fixed, prolonged, and rhythmic repetitive behaviors with an early onset and a fixed presentation pattern (DSM-5), can be confused with tics in animal models, though they are separated clinical entities. A discriminative method between these two motor phenotypes in animal models could increase *face validity* and help the development of more targeted therapeutic strategies. To achieve this point, a better understanding of the animals' behavioral spectrum along with a beter knowledge of tics' generating mechanisms are needed.

Finally, TS is classified as a neurodevelopmental syndrome, as it is typically diagnosed in childhood or adolescence, and tics show spontaneous and substantial reduction toward the end of the second decade of life in more than half of patients. However, so far, animal model research lacked the investigation of the way development affects the phenotype. Juvenile animal models could elucidate the impact of developmental mechanisms and importantly help the study of more effective and safer therapies for young patients.

## Author contributions

FR and EN equally contributed to the design, drafting, writing and revising of the work. AL contributed to the design, drafting, and critical revision under a clinical point of view. BH and TB contributed to the drafting, critical revision under a pre-clinical point of view, and final approval of the work.

## Funding

This work was supported by the European Commission Seventh Framework Programme (FP7-PEOPLE-2012-ITN), under the grant agreement n°316978.

### Conflict of interest statement

The authors declare that the research was conducted in the absence of any commercial or financial relationships that could be construed as a potential conflict of interest. The reviewer KRMV is a co-Topic Editor of the handling Editor for the Research Topic this article is published under. The authors and the handling Editor declared an ongoing co-authorship, and the handling Editor states that the process nevertheless met the standards of a fair and objective review.

## Footnotes

^1^http://www.ncbi.nlm.nih.gov/pmc/articles/PMC3894588/.

^2^http://www.pnas.org/content/suppl/2015/01/02/1419533112.DCSupplemental.

^3^http://www.frontiersin.org/Systems_Neuroscience/10.3389/fnsys.2013.00050/abstract.

^4^http://www.sciencedirect.com/science/article/pii/S0014488615000035.

## Abbreviations

ADHD: Attention Deficit/Hyperactivity Disorder
CSTC: Cortico-striato-thalamo-cortical circuit
DA: Dopamine
DOI, 2: 5-Dymethoxy-4-iodoamphetamine
DR: Dopaminergic Receptor
DSM-5: Diagnostic and Statistical Manual of Mental Disorders, Fifth Edition
GABA: Gamma-Aminobutyric acid
GPe: Globus pallidus externus
HDC: Histidine decarboxylase
KO: Knock-out
NTs: Neurotransmitters
OCD: Obsessive-Compulsive Disorder
PPI: Pre-Pulse Inhibition
PU: Premonitory Urge
TS: Tourette's Syndrome.
